# *Aedes aegypti* salivary gland extract alleviates acute itching by blocking TRPA1 channels

**DOI:** 10.3389/fphys.2023.1055706

**Published:** 2023-06-27

**Authors:** Anderson R. A. Cerqueira, Leandro Rodrigues, Silvia Abigail Coavoy-Sánchez, Simone A. Teixeira, Karla B. Feitosa, Erika Y. Taniguchi, Lucia R. Lopes, Antônio C. Cassola, Marcelo N. Muscará, Anderson Sá-Nunes, Soraia K. P. Costa

**Affiliations:** ^1^ Departamento de Farmacologia, Instituto de Ciências Biomédicas, Universidade de São Paulo, São Paulo, Brazil; ^2^ Departamento de Fisiologia e Biofísica, Instituto de Ciências Biomédicas, Universidade de São Paulo, São Paulo, Brazil; ^3^ Departamento de Imunologia, Instituto de Ciências Biomédicas, Universidade de São Paulo, São Paulo, Brazil; ^4^ Instituto Nacional de Ciência e Tecnologia em Entomologia Molecular, Conselho Nacional de Desenvolvimento Científico e Tecnológico (INCT-EM/CNPq), Rio de Janeiro, Brazil

**Keywords:** nonhistaminergic, skin, itch, sensory neurons, TRPA1, salivary gland, *Aedes* (Ae) *aegypti*, MrgprA3

## Abstract

*Aedes aegypti* (*Ae. aegypti*) saliva induces a variety of anti-inflammatory and immunomodulatory activities. Interestingly, although it is known that mosquito bites cause allergic reactions in sensitised hosts, the primary exposure of humans to *Ae. aegypti* does not evoke significant itching. Whether active components in the saliva of *Ae. aegypti* can counteract the normal itch reaction to injury produced by a histaminergic or non-histaminergic pathway in vertebrate hosts is unknown. This study investigated the effects of *Ae. aegypti* mosquito salivary gland extract (SGE) on sensitive reactions such as itching and associated skin inflammation. Acute pruritus and plasma extravasation were induced in mice by the intradermal injection of either compound 48/80 (C48/80), the Mas-related G protein-coupled receptor (Mrgpr) agonist chloroquine (CQ), or the transient receptor potential ankyrin 1 (TRPA1) agonist allyl isothiocyanate (AITC). The i.d. co-injection of *Ae. aegypti* SGE inhibited itching, plasma extravasation, and neutrophil influx evoked by C48/80, but it did not significantly affect mast cell degranulation *in situ* or *in vitro*. Additionally, SGE partially reduced CQ- and AITC-induced pruritus *in vivo*, suggesting that SGE affects pruriceptive nerve firing independently of the histaminergic pathway. Activation of TRPA1 significantly increased intracellular Ca^2+^ in TRPA-1-transfected HEK293t lineage, which was attenuated by SGE addition. We showed for the first time that *Ae. aegypti* SGE exerts anti-pruriceptive effects, which are partially regulated by the histamine-independent itch TRPA1 pathway. Thus, SGE may possess bioactive molecules with therapeutic potential for treating nonhistaminergic itch.

## 1 Introduction

The itch-scratch reflex is modulated by small-diameter sensory neurons in the dorsal root ganglia (DRG) and it is usually associated with inflammatory responses (Bartsch et al., 2019). This reaction is a protective sensory modality that triggers specific neural histaminergic and non-histaminergic pathways with a complex interplay between keratinocytes, immune cells, and cutaneous neurons (Koch et al., 2018; Moore et al., 2018). Whereas acute itch evoked by insect bites is relieved by scratching or antihistamine therapy and is mainly associated with biogenic amines released from mast cells, persistent (chronic) itching is commonly associated with chronic skin diseases, such as atopic dermatitis and, thus, difficult to control (Berger et al., 2013; Pereira et al., 2019; van Laarhoven et al., 2019). In this sense, patients with these conditions could benefit from additive antipruritic therapies.

The transient receptor potential (TRP) proteins are a superfamily of structurally related non-selective cation channels. One of its members, the transient receptor potential ankyrin 1 (TRPA1), mediates temperature sensing (Story et al., 2003; Moparthi et al., 2014) and is highly expressed in the terminal ends of sensory neurons throughout the body. It also functions as a chemosensor for pain-producing compounds and histamine-independent itch-signalling pathways (Lee et al., 2008; Liu et al., 2009; Wilson et al., 2011; Kemény et al., 2018; Liu et al., 2021). TRPA1 knockout mice exhibited a marked reduction of itching in response to selective agonists of Mas-related G protein-coupled receptor (Mrgpr) subtypes, such as chloroquine (CQ; MrgprA3) or BAM8-22 (MrgprC11). These receptors are expressed on C-fiber-sensitive neurons, thus reinforcing the role of TRPA1 receptor signalling in histamine-independent itch pathways (Wilson et al., 2011). Notably, the widely used secretagogue C48/80 can trigger Mas-related G protein-coupled receptor X2 (MRGPRX2), the mouse ortholog of MrgprB2, expressed in skin-resident mast cells, thus inducing pseudoallergies due to IgE-independent mast cell degranulation, the release of histamine and proteases, and thereby evoking inflammation and pruritus (Bradford, 1976; McNeil et al., 2015; Rodrigues et al., 2017; Dondalska et al., 2020; Yang et al., 2021). It was previously reported that murine MrgprA3 and human MrgprX1 are the predominant CQ receptors (Li et al., 2022).

*Aedes aegypti* (*Ae. aegypti*) saliva is a complex mixture of bioactive components with anti-hemostatic and immunomodulatory properties (Bissonnette et al., 1993; Ribeiro, 1995; Bizzarro et al., 2013; Barros et al., 2019). Together, these components contribute to female mosquitoes' blood acquisition and facilitate the transmission of several arboviruses (Jin et al., 2018; Sun et al., 2020). Due to these properties, *Ae. aegypti* saliva and its constituents have been prospected for compounds that can prevent or treat clinical conditions using experimental disease models (Sales-Campos et al., 2015; de Souza Gomes et al., 2018; Assis et al., 2021; Lara et al., 2021).

In addition to the various biological activities characterised in the *Ae. aegypti* saliva, there may still exist many others not yet to be identified and assessed. Knowing the expression profile of TRPA1 and its role as a pain chemosensor, we were interested in assessing the effect of SGE on TRPA1 by measuring TRPA1-mediated Ca^2+^ influx in hTRPA1-HEK293t cells and the *in vivo* effect of SGE on TRPA1 Allyl isothiocyanate (AITC) or MrgprA3 (CQ) receptor agonist-induced pruritus. Thus, the present study evaluated the effects of *Ae. aegypti* SGE on histaminergic and non-histaminergic itch induced in mice. We also evaluated if *Ae. aegypti* SGE acts on TRPA1 receptors using TRPA1-transfected HEK293t cells.

## 2 Methods

### 2.1 Animals

Male BALB/c mice and male Wistar rats, 7-10-week-old, were obtained from the institutional animal care facilities and housed in groups of five animals per cage under standard controlled conditions (22°C; 12/12 light/dark cycle) in ventilated racks (Alesco, Monte Mor, SP, Brazil) with free access to food and water. Experiments were approved by the Institutional Animal Care and Use Committee (CEUA-ICB/USP; protocol no. 33, page 85, book no. 02/2010), according to the guidelines of the Brazilian Council for Control of Animal Experimentation (CONCEA) and the Directive 2010/63/EU, combined with the Animal Welfare Act.

### 2.2 *Aedes aegypti* salivary gland extract

Mosquitoes were bred and maintained in an insectary at 26°C, 80% humidity, 12/12 light/dark cycle, and fed a 10% sucrose solution *ad libitum* (Maciel et al., 2014). Female mosquitos (5–7 days old) were anesthetised under low temperatures (4°C), sterilised in 70% alcohol, and immersed in 200 μL of phosphate-buffered saline (PBS). The mosquito heads were removed, and the salivary glands were dissected from the thorax under a microscope and transferred to a microtube containing 50 μL of cold PBS. The samples were sonicated and centrifuged (4 °C, 14,000 × *g*, 10 min) to remove particulate material (Bizzarro et al., 2013; Maciel et al., 2014). The supernatant was sterilised through a 0.22 μm nitrocellulose membrane filter (Millipore, Billerica, MA, United States). Protein concentration was measured using a NanoDrop 2000 (A_280_; Thermo Fisher Scientific, Wilmington, DE, United States). Aliquots of 2 mg/mL SGE were prepared and stored at −80°C until use.

### 2.3 Materials

3,3′-Dimethoxybenzidine dihydrochloride, urethane, HTAB (hexadecyltrimethylammonium bromide), Trypan blue, Toluidine blue, CQ (N-dimethyl-1,4-pentane diamine diphosphate), compound 48/80 (C48/80 N-methyl-p-methoxy phenethylamine), phthalaldehyde, AITC (Allyl isothiocyanate), 2-(1,3-Dimethyl-2,6-dioxo-1,2,3,6-tetrahydro-7H-purin-7-yl)-N-(4-isopropylphenyl)acetamide (HC030031), pyrilamine and Fura-2-AM were purchased from Sigma Chemical Co. (St Louis, MO, United States). Percoll was purchased from GE Healthcare (Uppsala, Sweden). Isoflurane (1-chloro-2,2,2-trifluoroethyl difluoromethyl ether) and sodium heparin were purchased from Cristália (Itapira, São Paulo, Brazil). Hydrogen peroxide (H_2_O_2_) and formaldehyde (CH_2_O) were purchased from Labsynth^®^ (Diadema, SP, Brazil).

### 2.4 Drug-induced scratching

Itch behaviour was evaluated as described (Costa et al., 2006; Rodrigues et al., 2017). C48/80 (10 μg/site), CQ (25 or 100 μg/site), AITC (20 mM reagent = 1 μmol/50 μL per site), or the corresponding vehicle (Tyrode solution) was intradermally (i.d.) injected, in a volume of 50 μL, alone or in combination with either *Ae. aegypti* SGE (1–10 μg/site) or the TRPA1 antagonist HC030031 (20 µg/site) or both. Mice were individually confined into an acrylic transparent box (12 × 20 × 17 cm) in a room fitted with a video recorder. The animals were acclimatised for 40 min for 2 days before the experiments. For animals showing a repeated number of scratching behaviour and a series of these movements, one bout of scratching was counted, and the number was expressed as absolute or percentage values determined in 30 min. In all experiments, the scratching behaviour was quantified in a blinded fashion.

### 2.5 Absorbance spectrum analysis of combined SGE and HC030031 solution

In order to evaluate the potential chemical interference between SGE and HC030031 (100, 200 and 400 μg/mL), the absorbance spectrum of increasing concentrations of SGE (50, 100 and 200 μg/mL) with and without increasing concentrations of HC030031 in 96-well microplates was measured on SpectraMax Plus 384 (Molecular Devices Corp, Sunnyvale, CA, United States) within the interval of 200–260 nm.

### 2.6 Assessment of plasma extravasation and myeloperoxidase activity

Shaved mice were anesthetised with urethane (2.5 g/kg; i.p.), and a volume of 100 μL of ^125^I–bovine serum albumin (^125^I-BSA, 0.037 MBq/mouse) was intravenously injected via the tail vein. Either C48/80 (10 μg/site) or vehicle (Tyrode) were i.d. injected alone or co-injected with SGE (1–10 μg/site) throughout six randomised skin sites (Rodrigues et al., 2017). Thirty minutes later, a blood sample was collected, and plasma was separated by centrifugation (10,000 × *g*, 10 min, 4°C). The skin injection sites were removed, and radioactivity present in these specimens was measured in a γ-counter (Packard Bioscience, Meriden, CT, USA). The plasma volume extravasated in the injected skin sites was calculated and expressed as μL of plasma per g of tissue or as a percentage of the control values (obtained with C48/80) alone.

In a separate set of experiment, mice were anaesthetised and i.d. injected with C48/80 (10 μg/site) or Tyrode in the presence or absence of SGE (1–10 μg/site). After 4 hours (4 h), animals were euthanised, and the injected sites were removed. Tissue MPO activity was measured as a marker of neutrophil infiltration into the skin tissue, as previously described (Rodrigues et al., 2017).

### 2.7 Immunohistochemistry for intercellular adhesion molecule-1 (ICAM-1) and vascular cell adhesion molecule-1 (VCAM-1) expression

Skin specimens i.d. injected with Tyrode, C48/80 (10 μg/site) alone or plus SGE (10 μg/site) were obtained and fixed in glass slides were heated (98 °C) for antigen retrieval in sodium citrate buffer (0.05% Tween-80, pH 6.0) and washed with PBS (pH 7.2). Following 30 min of incubation with 3% H_2_O_2_ in PBS (pH 7.2), the specimens were treated with 10% rabbit serum in PBS/10% BSA solution (1:1) for 1 h. The sections were overnight incubated with polyclonal antibodies anti-ICAM-1 (0.5 μg/mL); #AF796; R&D Systems, Minneapolis, MN, United States) or anti-VCAM-1 (2 μg/mL#AF643; R&D Systems) diluted in PBS/Tween 20 (0.3%). Following incubation with rabbit anti-goat IgG secondary antibody for 1 h (1 μg/mL]; at 21°C; Rockland Immunochemicals Inc., Limerick, PA, United States), vectastin ABC-kit (Vector Laboratories, Burlingame, CA, United States) was applied for 1 h for signal amplification and 3,3-diaminobenzidine, diluted in PBS containing 0.03% H_2_O_2_ (v/v), was utilised as the chromogen, yielding the overall brown colour. Images of three (ICAM-1) or six (VCAM-1) regions from both epidermis and dermis were randomly selected, and the stained area were acquired by optical microscopy (×200 magnification, LeicaDM 2500) and quantified using the Image-Pro Plus 4.5 software (Media Cybernetics, MD, United States). The images were calibrated using an image of a stage micrometer scale with the same optical set up and pre-established colour filter applied to quantify the positive areas. The ratio between the labelled (VCAM-1 or ICAM-1 immunoreactivity) area per total area analysed was given as stained area fraction in μm^2^. For each image a tissue area was defined and used to calculate the percentage (%) of positive area, based on the equation: (positive area/total tissue area) x 100.

### 2.8 Mast cell degranulation *in situ*

Mice were anaesthetised with isoflurane in oxygen, followed by i.d. injection with C48/80 (10 μg/site), Tyrode alone or in the presence of SGE (1 μg/site). After 30 min, the animals were submitted for euthanasia, and the i.d. injected skin sites were removed and soaked in 4% buffered formalin solution pH 7.4 for 24 h before being fixed in paraffin. Five micrometer-thick sections were mounted on glass microscope slides and stained with 1% toluidine blue working solution for 1–2 min at room temperature (22°C). The number of toluidine blue positive mast cells was quantified based on their morphological characteristics of intact or activated/degranulated cells by an investigator blinded to the treatment in randomly areas of ≅ 100 μm^2^ (Lidegran et al., 1996; Santos et al., 2014), using ten random fields of each 5 μm skin section, under optical microscopy (Leica DM2500; Basel, Switzerland), with a high-power objective lens (×40 and 100×). The number or corresponding percentage of degranulated mast cells was subsequently calculated. Complete cells were considered those with a well-defined contour and that are not in the process of changing shape and releasing the granule contents in the cytoplasm, whilst the activated/degranulated mast cells exhibited irregular contours and dispersed (metachromatic) granules.

### 2.9 Mast cell degranulation *in vitro*

A total number of 15 male rats were used in three independent experiments. Animals were exsanguinated under deep anaesthesia with inhaled isoflurane in oxygen. Mast cells were isolated from the peritoneal cavity and purified (95%) using a Percoll gradient. In a volume of 0.5 mL mast cell aliquots (4 × 10^5^ cells/mL), SGE was added in various concentrations (1–100 μg/mL) followed by C48/80 (1 μg/mL). After 15 min at 37 °C, samples were centrifuged (300×g, 4°C, 10 min) to determine the histamine released in the supernatant and residual histamine in the cell pellet. Samples were incubated with 300 μL of 1M NaOH and 1% phthalaldehyde (in methanol) for 4 min, followed by adding 15 μL of 3M HCl with vigorous agitation. Fluorescence was measured in a microplate reader (Synergy HT; Biotek, Winooski, VT, USA) at 360/450 nm (excitation/emission). Histamine concentrations were calculated from a histamine standard curve (prepared in 1M NaOH, range: 0.005–1.5 μg/mL). Results were expressed as % of histamine released [Histamine_released_/(Histamine_released_ + Histamine_cell lysate_) × 100].

### 2.10 HEK293t cell culture, TRPA1 transfection and western blot analysis

The human embryonic kidney cells (HEK 293), donated by Dr. Nancy Rebouças (ICB, University of São Paulo), were grown in Dulbecco’s Modified Eagle Medium (DMEM) supplemented with 10% fetal bovin serum, penicillin and streptomycin sulfate (1%; 37 °C, 5% CO2) and maintained in six-well culture plates until 80% confluence. The medium was removed, and the cells were incubated with Opti-MEM (#31985070, Invitrogen, Carlsbad, CA, United States) containing 5 µg of the hTRPA1 vector and 5 µg of pCMVSB100X in the presence of Lipofectamine 2000 for 8 h (37 °C, 5% CO_2_). After incubation, the medium was removed, and DMEM containing 10% SFB and G418 (500 μg/mL was added.

We expressed hTRPA1 in a TRPA1-negative cell line (HEK293t) to evaluate the inhibitory effect of SGE on TRPA1 receptor agonist AITC-induced increased intracellular Ca^2+^. For this, mammalian expression plasmid vectors hTRPA1 (# 100016279; Life Technologies, Carlsbad, CA, United States) and pCMVSB100X, a pT2/BH sleeping beauty transposon derived with ampicillin and neomycin resistance (# 26556; Addgene, Watertown, MA, United States), was provided by Dr Zoltan Sandor (Department of Pharmacology and Pharmacotherapy, University of Pecs, Hungary) and transfected into HEK293t cells using Lipofectamine 2000 (Pozsgai et al., 2017). A fluorescence assay was performed 4 h and 24 h after transfection.

Protein concentration was determined (Bradford, 1976), and TRPA1 expression was analysed using 20 μg of total protein by sodium dodecyl sulphate-polyacrylamide SDS-PAGE gel electrophoresis via Western blot device using specific electrophoresis buffer, accordingly (Laemmli, 1970). TRPA1-negative or TRPA1-positive cell line HEK293t (5x 10^6^ cell/mL) were centrifuged (10.000 rpm, 5 min at 4°C), and the resulting pellet resuspended in TRIS buffer (50 mM pH 7.4, Triton X-100 1%, sodium dodecyl sulfate 0.1%, phenylmethylsulfonyl fluoride 1 mM and protease inhibitor mixture 0.1%; Sigma, St. Louis, MO, United States) and assayed for TRPA1 expression. Both the primary antibody rabbit polyclonal anti-TRPA1 (1:1500; Novus Biologicals, CO, United States) and the corresponding HRP-conjugated secondary antibody (goat anti-rabbit; Bio-Rad Laboratoris, Inc, CA, United States) were used. The immunoreactive bands were detected by chemiluminescence in a ChemiDoc™ MP images system (Bio-Rad Laboratories, Inc, CA, United States). The molecular weights of the immunoreactive bands were determined using the ImageLab™ software by comparison of the electrophoretic mobilities with those of known molecular weight standard anti-β-Actin antibody (1:2000; Thermo Fisher Scientific, IL, United States).

### 2.11 Intracellular Ca^2+^

Transfected cells were incubated with Fura-2-AM at 37 °C. After 30 min, the cells were moved to a microscope, where they remained under a constant flow of Tyrode throughout the experiment. Tyrode buffer containing AITC (100 μM) or SGE (1 μg/mL) was alternated to evaluate fluorescence variations. During the experiments, the temperature was carefully controlled at 37°C (de Carvalho et al., 2014). The images were obtained using a Leica AF6000 inverted fluorescence microscope. For images with Fura-2-AM, a rotating excitation filter system of 340 and 380 nm was used. The emission was observed with a high-pass filter for wavelengths above 510 nm (2 Hz). The data presented as the 340/380 nm fluorescence ratio.

### 2.12 Data analysis and statistics

Data are expressed as the mean ± SD or median (interquartile range [IQR]) of n animals unless otherwise stated. A normality Shapiro–Wilk test was performed for each set of results. ANOVA, Sidak’s post-test, or Kruskal-Wallis, Dunn’s post-test were used to compare means between groups when the variable was in a normal or non-normal distribution, respectively. Values of *p* < 0.05 were considered significant. When required, Student’s t-test (two-tailed paired) were calculated. Data were analysed using GraphPad Prism Co. (Version 9.5.1, San Diego, CA, USA).

## 3 Results

### 3.1 Pruritus and skin inflammation

**FIGURE 1:**
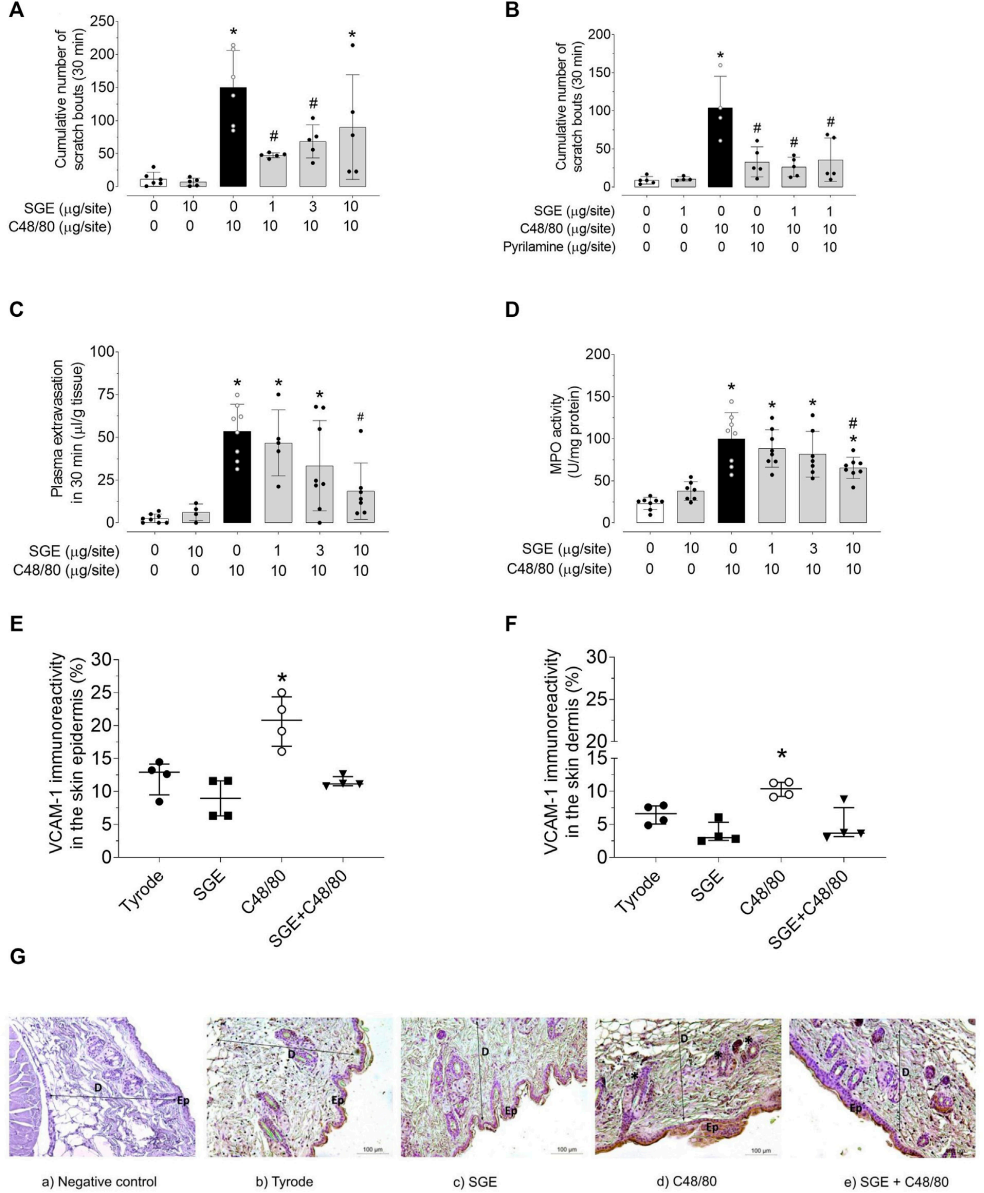
*Aedes aegypti* SGE downmodulates histaminergic responses triggered by compound 48/80. Bar scatter dot plot graphs showing itch evoked by C48/80 in the presence of different doses of SGE **(A)**, or in the presence of 1 μg/site dose of SGE alone and co-injected with the antihistamine pyrilamine **(B)**, effects of *Ae. aegypti* SGE on C48/80-induced plasma extravasation **(C)**, and C48/80-induced increased neutrophil influx measured by MPO activity **(D)**. Values are expressed as mean ± SD. *p < 0.05 *vs.* “Vehicle or *Ae. aegypti* SGE group”; ^#^ *p* < 0.05 vs. “C48/80” group (*n* = 4–8). Quantitative immunoreactivity staining analysis showing the % of VCAM-1 positive epidermal and dermal area of C48/80 (10 μg/site)-injected skin treated and untreated with SGE (10 μg/site) represented by the column scatter dot plot graphs **(E)** and **(F)**, respectively. Data are expressed as median with interquartile range (*n* = 4 mice/group). *p < 0.05 vs *Ae. aegypti* SGE group. Panel G shows representative images of VCAM-1 immunohistochemical staining differences (* asterisks show DAB-positive signal) in the epidermis and dermis of mouse dorsal skin i.d. injected with Tyrode **(B)**, SGE **(C)**, C48/80, **(D)** and C48/80 + SGE **(E)**. Panel **(Ga)** shows the negative control specimen (skin without primary antibody). Double ended arrows indicate dermal-epidermal junction, Ep = epidermis, D = dermis. DAB, hematoxylin counterstain, ×100 magnification.

Figure 1A shows that itch behaviour was effectively triggered in the animals by i.d. injection of C48/80 in the dorsal skin. The co-injection of SGE significantly reduced this response at the lowest and intermediate doses (1 and 3 μg/site). The histamine receptor antagonist pyrilamine (10 μg/site) reduced C48/80-induced pruritus, and the presence of co-injected SGE could not further inhibit the pruritus response (Figure 1B).

SGE, at 10 μg/site, but not at 1 and 3 μg/site, significantly prevented C48/80-induced plasma extravasation by ∼50% (Figure 1C). SGE alone did not evoke any plasma extravasation compared to Tyrode (Figure 1C).

C48/80 (10 μg/site) promoted a significant increase in tissue MPO compared to vehicle, and co-injection with SGE at the highest dose (10 μg/site) significantly decreased MPO activity (Figure 1D). SGE alone did not induce increased MPO activity compared to Tyrode (Figure 1D).

Immunohistochemistry analyses revealed that VCAM-1 (Figures 1E,F and G), but not ICAM-1 (Supplementary Figure S1A, B; Supplementary Table S1) expression was significantly upregulated in the epidermis and dermis of mice dorsal skin by the i.d. injection of C48/80 (10 μg/site) 4 h later. When co-injected with SGE, C48/80-induced VCAM-1 expression did not significantly differ from SGE- or Tyrode-induced response (Figures 1E,F and G; Supplementary Figure S1A, B; Supplementary Table S1).

### 3.2 Mast cell stabilisation *in situ and in vitro*

**FIGURE 2:**
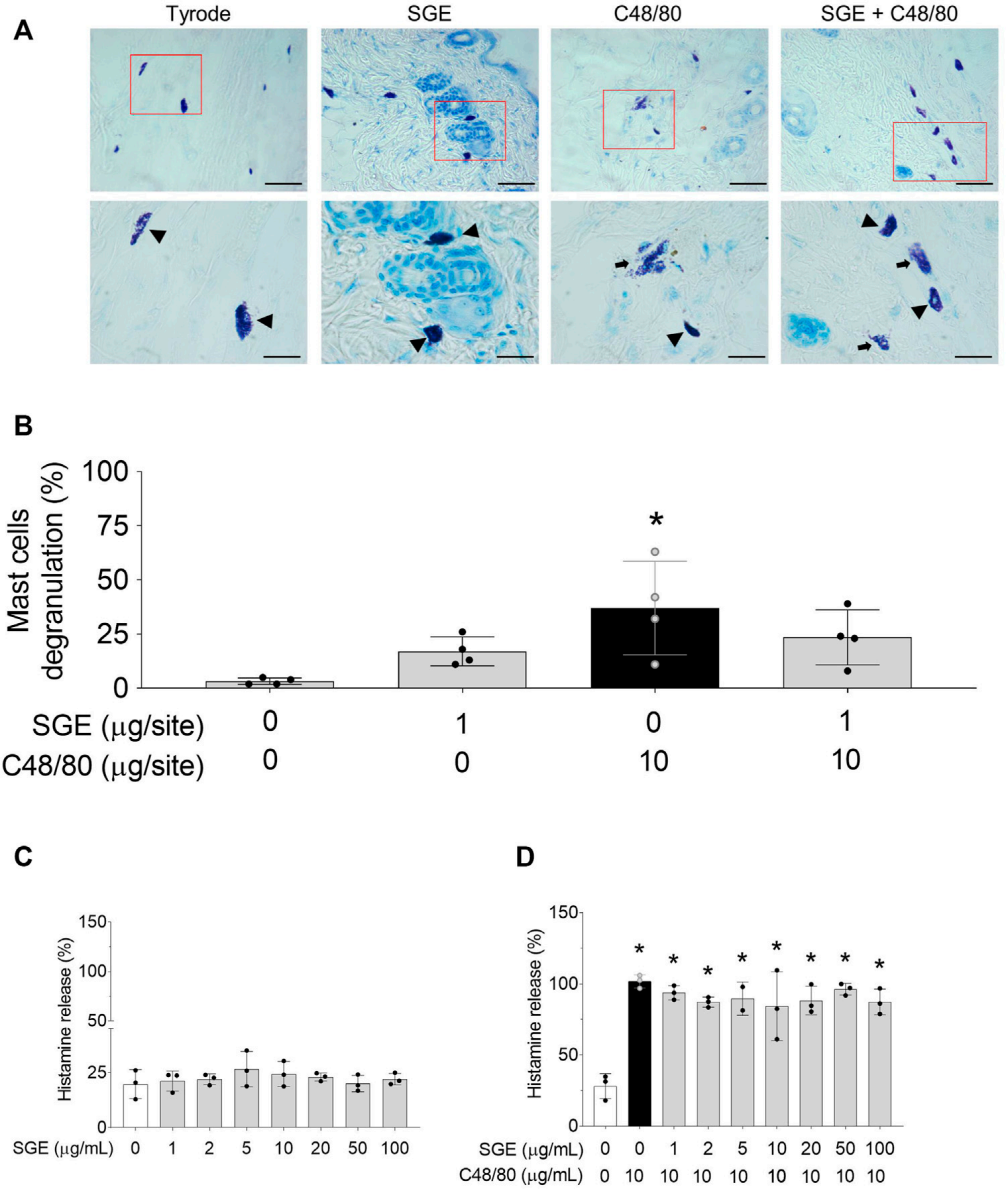
Effects of *Aedes aegypti* SGE on mast cell degranulation induced by C48/80. Panel **(A)** illustrates representative images of intact (arrowhead) and degranulated mast cells (arrow) in mice´s skin *in situ* (by the colouration of toluidine blue) following 30 min i.d. injection of Tyrode, SGE, C48/80 and C48/80 + SGE, respectively, on the magnification of ×400 (top images) and 1000x (bottom images), Bar = 50 μm. Panel **(B)** shows the corresponding percentage of degranulated mast cells counted in random fields of the observed mice skin tissue *in situ*. Data are expressed as mean ± SD for n = 4 mice/group. *p < 0.05 vs. “Vehicle” group. Panels **(C, D)** show the effect of increasing concentrations of SGE on the percentage of histamine released *in vitro* from rat peritoneal mast cell following 15 min incubation with vehicle or C48/80, respectively. Data presented as scatter plot expressed as mean ± SD are representative of three independent experiments for a total n = 15 rats. *p < 0.05 vs. “Vehicle” group. One-way ANOVA (Sidak’s multiple comparisons test).

Mast cell staining with toluidine blue was not triggered by the i.d. injection of vehicle or Ae. aegypti SGE in the mouse dorsal skin; however, an i.d. injection of C48/80 promoted significative *in situ* mast cell degranulation compared to the vehicle or SGE alone (Figures 2A, B; Supplementary Table S2). Co-injection of C48/80 with SGE did not significantly reduce the amount of degranulated mast cells (Figures 2A, B; Supplementary Table S2). The *in vitro* incubation of mast cells obtained from the rat peritoneal cavity with increasing concentrations of SGE did not significantly promote histamine release from these cells compared with the control (Figure 2C). C48/80 significantly induced mast cell histamine release compared to the control group, which was not affected by pre-treating the cells with different SGE concentrations (Figure 2D).

### 3.3 TRPA1 signalling mediates the anti-pruriceptive properties of SGE

**FIGURE 3:**
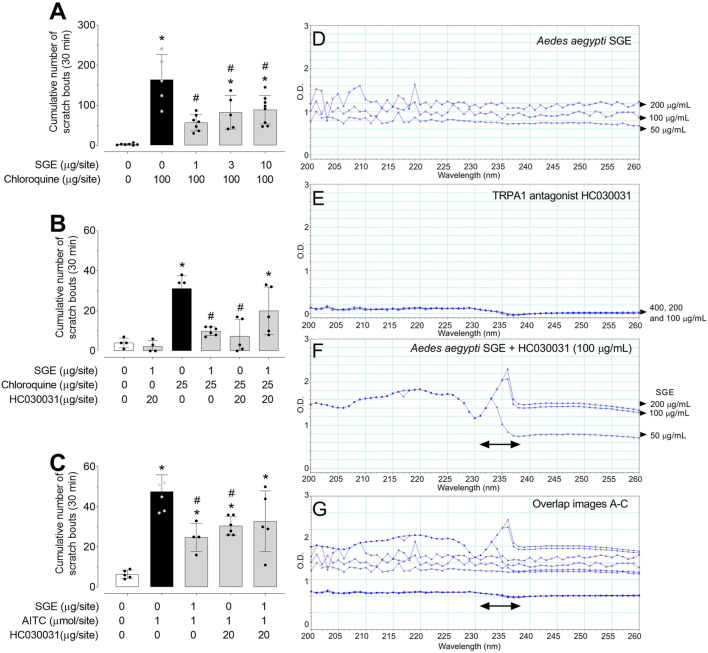
*Aedes aegypti* SGE downmodulates non-histaminergic itch caused by TRPA1 interaction evaluated during 30 min. Itch evoked by chloroquine in the presence of increasing doses of SGE **(A)**. Effect of SGE and/or TRPA1 antagonist HC030031 in chloroquine-induced itch **(B)**. Effect of SGE and/or TRPA1 antagonist HC030031 in AITC-induced itch **(C)**. Data are expressed as mean ± SD. *p < 0.05 vs. “Vehicle” group; ^#^ *p* < 0.05 vs. “chloroquine” or “AITC” group (n = four to eight mice). Panels **(D, E)** show the absorbance spectrum of three different concentrations of SGE (50, 100 and 200 μg/mL) or HC03003 (100, 200 and 400 μg/mL), respectively measured in the 200–260 nm range. Panel **(F)** shows the spectra of three individually concentrations of SGE (50, 100 and 200 μg/mL) co-incubated with HC030031 (100 μg/mL), in which an important change in the absorbance spectrum was observed in the 230–237 nm interval compared with either compound alone. Panel **(G)** shows overlap images for all testing substances. One-way ANOVA (Sidak’s multiple comparisons test).

CQ-induced intense pruritus was significantly decreased when SGE was co-injected at all the tested doses (Figure 3A). CQ (25 μg/site)-induced moderate pruritus was also inhibited by the TRPA1 antagonist HC030031 (20 µg/site; Figure 3B), showing the involvement of TRPA1 channels in the CQ response. Likewise, the AITC-evoked scratching behaviour was effectively antagonised by HC030031 (20 µg/site) and the *Ae.* ae*gypti* SGE co-injection (1 µg/site; Figure 3C, respectively). In the presence of SGE and HC030031, the itching behaviour induced by either agonist was not markedly affected. Of note, the combination of SGE and HC030031 did not promote itch (Figure 3B).

The spectra measured in the 200–260 nm range of SGE and HC030031, individually or co-incubated, showed that combined SGE and HC030031 changed the absorbance spectrum in the 230–237 nm interval compared with either compound alone (Figures 3D–G).

**FIGURE 4:**
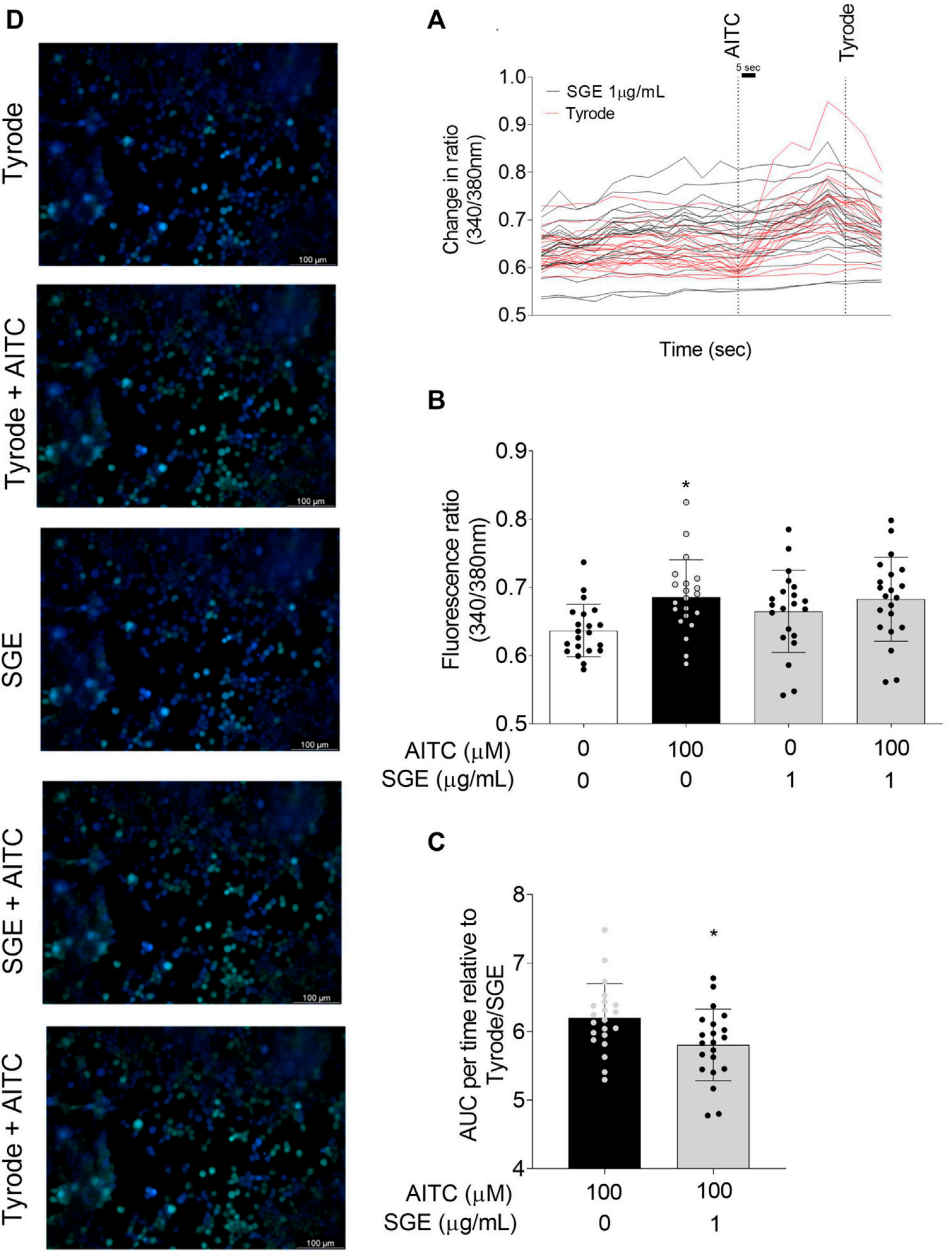
*Aedes aegypti* SGE attenuates TRPA1 receptor agonist AITC-induced Ca^2+^ mobilization in hTRPA1-HEK293t cells. Panel **(A)** shows representative scan lines displayed horizontally of continuously Ca^2+^ changes in relation to the time (sec) in responses to SGE (1 μg/mL), SGE +100 μM AITC, and Tyrode +100 μM AITC in TRPA1-HEK293t cells loaded with Fura-2-AM. Panel **(B)** shows corresponding fluorescence ratio (calcium changes) as mean ± SD from 3 independent experiments in responses to SGE (1 μg/mL), SGE +100 μM AITC, and Tyrode +100 μM AITC in TRPA1-HEK293t cells loaded with Fura-2-AM (n = 21 cells). *p < 0.05 vs. “Control” group. Fura-2 fluorescence signals are presented as the 340/380 nm ratio. Panel **(C)** demonstrates values as mean ± SD of the area under the curve (AUC) per time relative to Tyrode/SGE of calcium responses evoked by AITC (100 μM) in hTRPA1-HEK293t cells. *p < 0.05 vs. “Vehicle” group. One-way ANOVA (Sidak’s multiple comparisons test; panel **(A)** or Student’s t-test was used (panel **(B)**). The left **(D)** panel containing five images demonstrates the fractional fluorescence recorded from hTRPA1-HEK293t cells loaded with Fura-2-AM and scanned at a rate of ≅ 3 s per image in response to different stimuli. Transient events were recorded before (top image; basal fluorescence of the cells treated with vehicle Tyrode). The second from the top image illustrates the dynamic changes in intracellular Ca^2+^ measured by changes in the intensity of Fura-2-AM fluorescence (green colour) following the addition of AITC (peak time≅ 300 s). After washing out the cells with Tyrode, the third and fourth images illustrate reduced changes in fluorescence intensity after the addition of SGE alone (time ≅ 480 s) and together with AITC (time≅ 510 s), respectively. The bottom image illustrates intracellular Ca^2+^ increases in response to the new addition of AITC (time≅ 780 s) following a new washing session of the cells to assess viability. Non-responsive cells are observed in blue.

The WB analysis revealed immunoreactive bands in TRPA1-positive cells but not in negative hTRPA1-HEK293 cells, consistent with TRPA1 (≅ 105 kDa) and β-actin MW (≅ 33 kDa; Supplementary Figure S2). Given the requirement for TRPA1 mediating calcium response *in vitro*, we showed that hTRPA1-HEK293t cells stimulated with AITC exhibited significant calcium increment (mean fluorescence ratio 340/380 nm), when compared to hTRPA1-HEK293t cells stimulated with Tyrode (Figures 4A, B, D; Supplementary Figure S3A, B); but not with SGE (Figures 4A–D). Fluorescence changes in Ca^2+^ were taken continuously on the same cell population (Supplementary Figure S3; Supplementary Table S3). When analysed as the area under the curve (AUC) in relation to the vehicle, the treatment with SGE significantly attenuated AITC-induced calcium increase in hTRPA1-HEK293t cells (Figures 4C,D).

## 4 Discussion and concluding remarks

Pruritus is the most frequent symptom in dermatologic clinics and is among the 50 most prevalent conditions worldwide (Roh et al., 2021). The incidence of chronic pruritus varies among different studies, but it seems especially common among the elderly (Yalçin et al., 2006). The local itching sensation is intimately associated with touch, pain, and insect bites. However, at least for *Ae.* a*egypti* bites, pruritus is instead associated with hypersensitivity reactions since non-sensitive individuals did not report an itch sensation within 5 minutes of mosquito exposure (Conway, 2021). As opposed to the excessive inflammatory skin reaction observed in sensitised mice (Henrique et al., 2019), *Ae. aegypti* saliva and salivary preparations are associated with anti-inflammatory responses in various experimental conditions (Bissonnette et al., 1993; Surasombatpattana et al., 2012; Barros et al., 2019; Assis et al., 2021; Lara et al., 2021). This result suggests that *Ae. aegypti* saliva *per se* has no components capable of inducing acute pruritus, and the pruritus symptom may occur later in the bite site due to an allergic reaction triggered by immunoglobulin E (IgE) antibodies resulting from the host’s previous sensitisation (Barros et al., 2016).

Several groups, including our own, showed that histamine or C48/80 injected in rodent skin promotes pruritus, and this effect is abolished by histamine receptor antagonists and mast cell stabilisers (Rodrigues et al., 2017; Thangam et al., 2018). We showed that co-injection of SGE with C48/80 reduced C48/80-induced pruritus but not in a dose-dependent manner. Of note, in the absence of stimulation, the injection of *Ae. aegypti* SGE promoted a similar response to that evoked with Tyrode (control). *Ae. aegypti* saliva comprises a complex mixture of bioactive components with anti-hemostatic and immunomodulatory properties, with therapeutic potential both *in vivo* and *in vitro* studies. Among these molecules, those from the D7 family can bind a variety of vasoactive components, such as biogenic amines, including histamine and serotonin (Calvo et al., 2006; Martin-Martin et al., 2020; Martin-Martin et al., 2021).

Mast cell degranulation induced by C48/80 includes the interaction of the molecule with Gi_2_ and Gi_3_ proteins present in the cell membrane, stimulating phospholipase C-dependent signalling, promoting the synthesis of second messengers and increased intracellular Ca^2+^, and favouring the granule breakdown and the release of histamine release and other cell contents (de Vasconcelos et al., 2011; Yang et al., 2016). Accordingly, C48/80-induced plasma extravasation and increased MPO activity, which is an indirect marker for neutrophil presence in the mouse dorsal skin. The co-injection with SGE resulted in a trend to dose-dependently decrease C48/80-induced plasma extravasation, but only the highest dose of SGE reduced the skin plasma extravasation significantly.

SGE, at the highest dose, also reduced C48/80-increased MPO activity in the tissue. Our hypothesis is that bioactive component(s) present in the *Ae. aegypti* SGE could prevent neutrophil recruitment by acting on mast cells (i.e., stabilising the mast cell membrane–preventing its degranulation – and/or interfering with the H1 receptor-dependent responses). This hypothesis was examined histologically *in situ* by staining tissue sections with toluidine blue, which binds to the glycosaminoglycans in the mast cell granules (Sridharan & Shankar, 2012), and by *in vitro* histamine determinations. SGE did not significantly change the integrity of mast cells *in situ* in the mouse dorsal skin i.d. injected with the C48/80 or the histamine content *in vitro* in the presence or absence of C48/80. Our data corroborate previous findings, showing that *Ae. aegypti* SGE could not change antigen-dependent mast cell degranulation, although it decreased the TNF-α production by these cells (Bissonnette et al., 1993).

Because *Ae. aegypti* SGE did not change mast cell phenotype or histamine release, we next evaluated whether the decreased MPO activity could be related to the modulation of adhesion molecules in the tissue. In this sense, ICAM-1 and VCAM-1 are actively involved in cell adhesion to the endothelium and transmigration to the tissues. The immunohistochemical analysis of skin showed no changes in ICAM-1 expression, but a significant increase in (%) VCAM-1 expression was detected in both the dermis and epidermis of mice receiving C48/80 alone, while its co-injection with SGE did not significantly differ from control levels. Mast cell-dependent ICAM-1 expression has been shown in chronic cutaneous conditions, such as psoriasis and atopic dermatitis (Ackermann & Harvima, 1998), but it was never reported at earlier time points. Regarding VCAM-1 expression, no study has evaluated its expression following C48/80 injection in the skin *in situ*. We hypothesised that 4 h after C48/80 injection (as assessed by our study) is not enough time to observe marked changes in the expression of these molecules. In agreement, an increase of ICAM-1 and VCAM-1 was observed *in vitro* in endothelial cells cultured with intact mast cells or C48/80-degranulated mast cells, but only after 16 h of co-incubation (van Haaster et al., 1997). Although discrete, the VCAM-1 results reinforce the findings observed for MPO activity since VCAM-1 is dependent on NF-κΒ activation (Nourshargh, 1993). Thus, our results indicate that *Ae. aegypti* SGE may affect endothelial cells, in line with a previous study (Schmid et al., 2016).

Sensory neurons expressing Mrgprs also regulate histamine-independent pruritus (Wilson et al., 2011). Due to the critical role of various proteases, TRPs, and Mrgprs in skin homeostasis and the pathophysiology of pruritus (Rajka, 1967; Steinhoff et al., 2003; Reddy et al., 2015; Coavoy-Sánchez et al., 2016), the co-participation of these receptors in the anti-pruriceptive effects of SGE was assessed. CQ is commonly used to prevent or treat malaria, but, as a side effect, produces intense itching in humans (Sowunmi et al., 1989; Sowunmi et al., 2001) or in mice when i.d. injected (Liu et al., 2009; Haddadi et al., 2020). This symptom cannot be alleviated by antihistamine drugs, reinforcing the role of a histamine-independent pathway (Li et al., 2022). Accordingly, co-injection of *Ae. aegypti* SGE significantly inhibited in a non-dose-dependent manner, the intense pruritus evoked by a high CQ dose. Curiously, the simultaneous i.d. injection with the TRPA1 antagonist HC030031 did not further enhance *Ae. aegypti* SGE anti-pruriceptive effect on CQ-induced pruritus and instead, it reversed the anti-pruritus effect of *Ae. aegypti* SGE against CQ-induced mild pruritus.

With this in mind, we hypothesise that TRPA1 is somehow essential to the signalling pathways that regulate the *Ae. aegypti* SGE anti-pruriceptive effect against CQ-induced pruritus. In agreement, in cultured DRG neurons, MAS-related GPCRs, MrgprA3 and MrgprC11, modulate the function of TRPA1 (Wilson et al., 2011; Lieu et al., 2014), which is activated by its agonist AITC. Moreover, at 1 μmol, AITC promoted scratching behaviour when i.d. injected in the mouse dorsal skin (Coavoy-Sánchez et al., 2016) or into the mouse nape (Liu et al., 2021), while in the cheek model, doses of 1–4 μmol evoked both wiping and scratching behaviour (Esancy et al., 2018; Han et al., 2018; Liu et al., 2021). Therefore, pruritus and nocifensive behaviour vary according to the dose and the site of TRPA1 agonist injection. Indeed, differences in TRPA1 expression can be found in the origins and extent of afferent fibers whose cell bodies reside in the DRG and trigeminal ganglion in the cutaneous area (Dong and Dong, 2018; Tobori et al., 2021).

Herein, the i.d. injection of TRPA1 agonist AITC in the mouse dorsal skin promoted itching behavior, which was significantly attenuated by the co-injection with *Ae. aegypti* SGE. However, the simultaneous injection with HC030031 neutralized SGE anti-pruriceptive effect against AITC-induced pruritus, thus suggesting that SGE (or some of its components) might typically act as partial agonist for TRPA1 receptors or perhaps, giving the biochemical complexity of SGE, an optimum range of action is reached for each situation due to the presence of molecules with additive, synergic or even opposite effects that might sometimes occlude each other, which may be the case here. It should also be noted that *Ae. aegypti* saliva contains kratagonists, scavenger molecules that interact with physiological effectors (Andersen & Ribeiro, 2017).

In fact, the absorption measurement of SGE and HC030031 combined solutions revealed notable changes in the 230–237 nm interval compared with either agent alone, indicating a potential antagonistic interaction between SGE and HC030031, which might explain the loss of efficacy on CQ- or AITC-induced pruritus. Furthermore, the presence of protease inhibitors has been revealed by transcriptome and proteome in *Ae. aegypti* salivary glands (Almeras et al., 2010; Ribeiro et al., 2016). These inhibitors may disrupt the cleavage of the amino-terminal region of the receptors and the activation of TRPA1 required for signal transduction (Coavoy-Sánchez et al., 2016; Derouiche et al., 2021). As suggested for other arthropod vectors (Sá-Nunes & Oliveira, 2021), the potential immunobiological properties of *Ae. aegypti* saliva have been successfully evaluated in several inflammatory models (Sales-Campos et al., 2015; de Souza Gomes et al., 2018; Assis et al., 2021). In some cases, the salivary molecules responsible for such activities have been elucidated. For example, one protein of the D7 family (scavengers of biogenic amines) was able to inhibit dengue virus infection (Conway et al., 2016); the peptide AeMOPE-1 displays many activities in macrophages and ameliorates experimental colitis (Lara et al., 2021); and many salivary proteins can interact with human receptors involved in immune responses (Gavor et al., 2022).

Corroborating the findings *in vivo*, we showed that increased TRPA1-mediated calcium influx (measured by the Fura-2-AM) assay in TRPA1-hHEK293t cells is significantly reduced by the treatment with *Ae aegypti* SGE, thus supporting the suggestion that bioactive components present in *Ae. aegypti* SGE can act, at least partially, on non-histaminergic (TRPA1) pathways of pruritus.

Our findings show the potential of salivary components of *Ae*. *aegypti* as a prospective source of bioactive molecules that may serve as templates for developing new compounds to prevent or treat skin conditions involving the histamine-independent itch TRPA1 pathway.

## Data Availability

The raw data supporting the conclusion of this article will be made available by the authors, without undue reservation.

## Supplementary material

Supplementary Figure S1 Quantitative immunoreactivity staining analysis showing the percentage of ICAM-1 positive dermal area in the mouse dorsal skin i.d. injected with C48/80 (10 mg/site) in the absence and presence of SGE. Data are represented by the column scatter dot plot graphs **(A)**, and expressed as median with interquartile range (n = 4 mice/group). Panel **B** shows representative images containing scarce ICAM-1 expression in the dermis of mouse dorsal skin i.d. injected with Tyrode **(B)**, SGE **(C)**, C48/80, **(D)** and C48/80 + SGE **(E)**. Panel **G(a)** shows the negative control specimen (skin without primary antibody). Arrows show hair follicles, Ep = epidermis, D = dermis. DAB, hematoxylin counterstain, 100× magnification.

Supplementary Figure S2 Representative Western Blot of TRPA1 expression in both hTRPA1-positve (transfected) and hTRPA1-negative (non-transfected) HEK293t (5 × 10^6^) cells. Immunoreactive bands are correspondent to approximately 42 kDa and 127 kDa for β-actin and TRPA1, respectively. hTRPA1-negative cells (non-transfected) or cells exposed to empty plasmid did not exhibit TRPA1 expression.

Supplementary Figure S3
*Ae aegypti* SGE reduced hTRPA1-mediated receptor mobilization of intracellular calcium in TRPA1-HEK293t cells in response to TRPA1 agonist AITC. Representative individual traces showing intracellular calcium changes in responses to SGE (1 μg/mL), SGE + 100 μM AITC, and Tyrode + 100 μM AITC in TRPA1-HEK293t cells loaded with Fura-2-AM (panel **A**). Panel **B** shows corresponding scan line displayed of continuously calcium changes (mean ± SD) from 5 independent experiments in responses to SGE (1 μg/mL), SGE + 100 μM AITC, and Tyrode + 100 μM AITC in TRPA1-HEK293t cells (n = 21) loaded with Fura-2-AM. Fura-2 fluorescence signals are presented as the 340/380 nm ratio.
