# Supplementary figures and images for: Aedes aegypti salivary gland extract alleviates acute itching by blocking TRPA1 channels
Source: Front Physiol. 2023 Jun 27;14:1055706. doi: 10.3389/fphys.2023.1055706 (PMC10333701; doi:10.3389/fphys.2023.1055706)

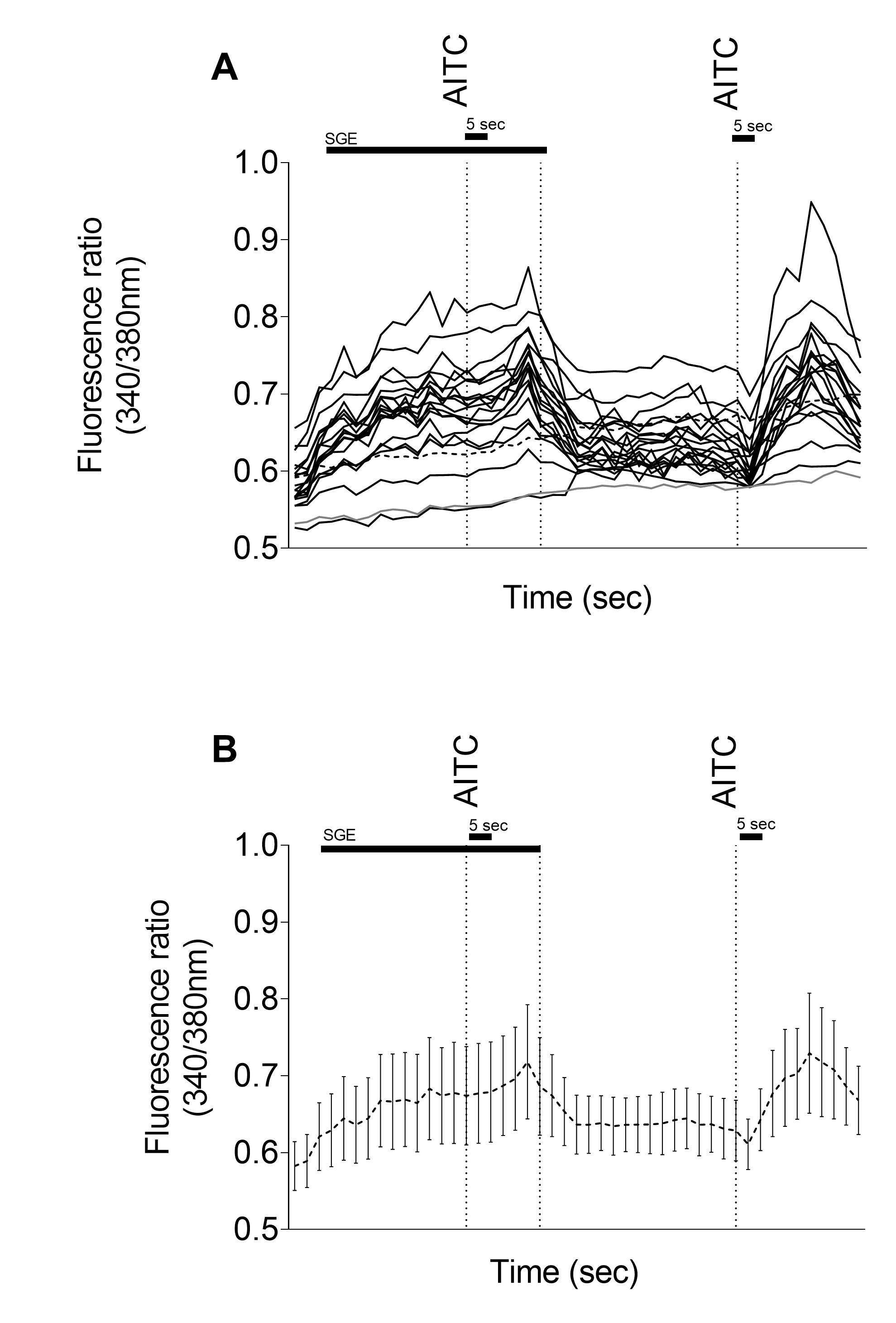

Supplement: Supplementary file 1 [file Image3.JPEG]

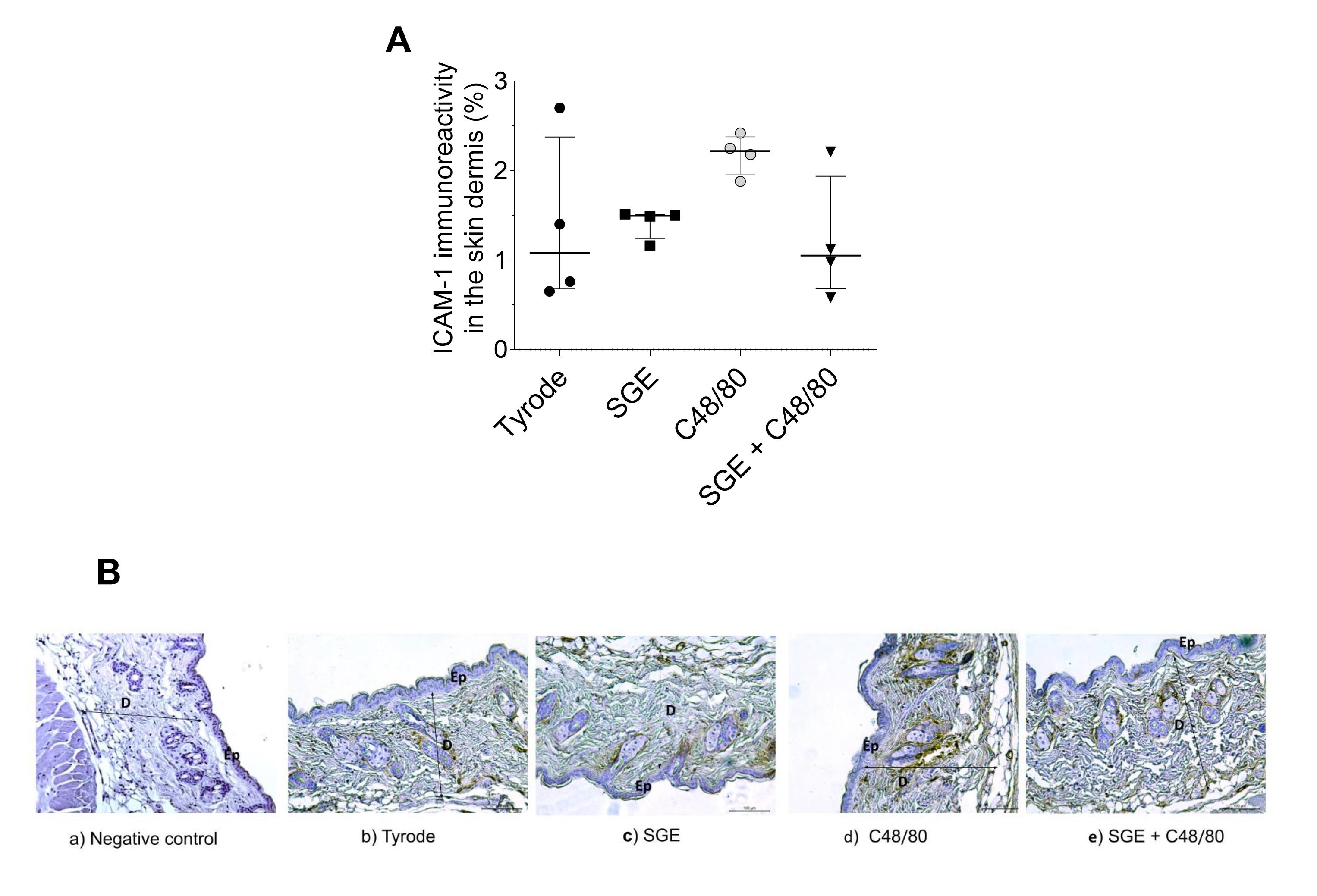

Supplement: Supplementary file 2 [file Image1.JPEG]

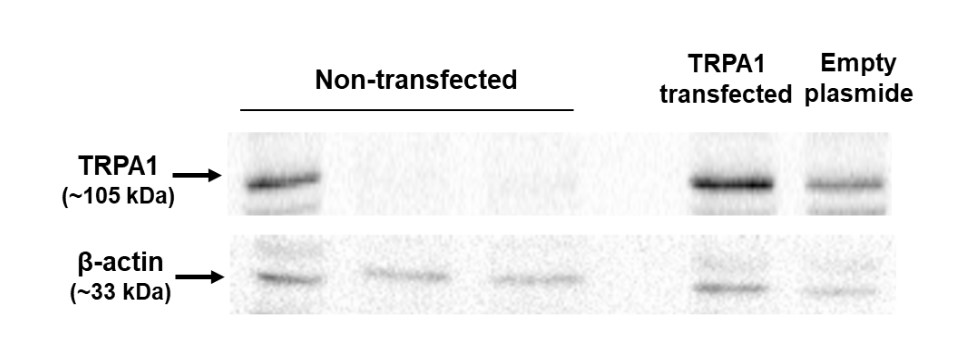

Supplement: Supplementary file 3 [file Image2.JPEG]
