# Supplementary material for: Aedes aegypti salivary gland extract alleviates acute itching by blocking TRPA1 channels
Source: Front Physiol. 2023 Jun 27;14:1055706. doi: 10.3389/fphys.2023.1055706 (PMC10333701; doi:10.3389/fphys.2023.1055706)
Supplement: Supplementary file 4 [file DataSheet1.PDF]

Supplemental Tables

Table 1 Summarizes the effect of *Ae. Aegypti* salivary gland extract (SGE) on the immunohistochemical staining for VCAM-1 and ICAM-1 in mouse dorsal skin i.d. injected with C48/80. The ratio between the labelled area (immunoreactivity) per total area analysed is given as stained area fraction in μm^2. Data are expressed as mean ± SD (n= 4) for total and DAB-positive epidermal and dermal surface area in response to i.d. injection of Tyrode, SGE, C48/80, or C48/80 co-injected with SGE. No detection of ICAM-1 was observed in the epidermis in response to i.d. injection of test agents. The immuno-positive area (μm^2) was assessed by Image-Pro Plus 4.5 software. The normal distribution of VCAM-1 or ICAM-1 expression was investigated by Shapiro-Wilk test. Differences between groups were analysed by ANOVA followed by Sidak's multiple comparisons post-test, or by non-parametric statistics applying the Kruskal-Wallis test, followed by Dunn's test for multiple comparisons when required. *P<0.05 *vs.* Tyrode or SGE, and #P<0.05 *vs.* C48/80.

	**Adhesion molecule positive area in mm^2 (Mean ± SD; n=4)**					
	**VCAM-1 / Epidermis**		**VCAM-1 / Dermis**		**ICAM-1 / Dermis**	
**Groups**	**Total area**	**Positive area**	**Total area**	**Positive area**	**Total area**	**Positive area**
Tyrode	21232±9171	2624±1373	132155±16425	8677±2772	124640±26964	1625±863
SGE	16547±4157	1526±791	107418±21039	3752±1629	152481±23532	2137±393
C48/80	20682±4762	4235±1221*	133855±7486	13669±2463*	121879±25932	2658±674
SGE + C48/80	18263±4454	2086±581	137058±9136	6546±3741#	133589±16787	1577±717

Table 2 Evaluation of mast cell count in response to intradermal injection of C48/80 in the mouse dorsal in the absence and presence of *Ae. Aegypti* salivary gland extract (SGE). Intact, activated and total number of mast cells for each treatment were counted in an area of 100 µm² in 5 µm sections from four animals. Data as absolute values and percentage are described as mean ± SD of intact, activated/degranulated and total mast cell for n = 4 mice per group. The normal distribution of mast cell variable was investigated by Shapiro-Wilk test, and differences between groups were analysed by ANOVA followed by Sidak's multiple comparisons post-test, or by non-parametric statistics applying the Kruskal-Wallis test, followed by Dunn's test for multiple comparisons. *P <0.05 vs Tyrode.

**Mast cell type**	**Groups (Mean ± SD)**			
	**Tyrode**	**SGE**	**C48/80**	**SGE + C48/80**
Intact	0.58 ± 0.10	0.51 ± 0.08	0.25 ± 0.10*	0.39 ± 0.14
Activated/degranulated	0.05 ± 0.02	0.09 ± 0.08	0.24 ± 0.12*	0.14 ± 0.04
Values in (%)	8.80 ± 3.53	14.37 ± 11.45	48.57 ± 16.18*	28.31 ± 8.54
**Total**	0.63 ± 0.09	0.60 ± 0.14	0.49 ± 0.11	0.53 ± 0.20

Table 3. Effects of *Ae. Aegypti* salivary gland extract (SGE) on TRPA1 (AITC)-induced changes in [Ca^{2+}] in human embryonic kidney cells (HEK 293) expressing TRPA1.We have monitored changes in [Ca^{2+}] in TRPA1 transfected HEK 293 cells using Fura 2-AM. We then monitored oscillation of [Ca^{2+}] in the cells in response to vehicle (Tyrode) or test agents (SGE and AITC). Cell images were taken continuously during the protocol on the same cell population in 10 min intervals. The Ca^{2+} ion fluorescence was observed for 40 sec, basal florescence change was observed after 6 sec, and maximum fluorescence change was observed after 36 sec. Afterwards, SGE + AITC (100 µM) was added to the mixture at 42 sec, change in basal

fluorescence was observed after 9 sec and the maximum fluorescence was observed after 15 sec. AITC + SGE mixture was then washed with Tyrode, and AITC (100 µM) was finally repeated, and the Ca^{2+} ion fluorescence was observed for 30 sec. Each data point in the columns represents the time interval that the change in fluorescence occurred. n=21 cells.

**Intervals - Fluorescence changes in ion Ca^{2+}**					
**Treatment**	Load	Change in basal fluorescence	Maximum fluorescence	Δ Basal changes Load	Δ Maximum fluorescence
SGE	0	6	36	6	36
SGE + AITC	42	51	57	9	15
AITC	108	111	126	3	18
